# The histone H2B Arg95 residue efficiently recruits the transcription factor Spt16 to mediate Ste5 expression of the pheromone response pathway

**DOI:** 10.1038/s41598-023-37339-y

**Published:** 2023-06-22

**Authors:** Abdallah Alhaj Sulaiman, Reem Ali, Dindial Ramotar

**Affiliations:** Division of Biological and Biomedical Sciences, College of Health and Life Sciences, Hamad Bin Khalifa University, Qatar Foundation, Education City, P.O. Box: 34110, Doha, Qatar

**Keywords:** Cell biology, Drug discovery

## Abstract

In yeast *Saccharomyces cerevisiae*, the immunosuppressant rapamycin inhibits the TORC1 kinase causing rapid alteration in gene expression and leading to G_1_ arrest. We recently reported the isolation and characterization from the histone mutant collection of a histone H2B R95A mutant that displays resistance to rapamycin. This mutant is defective in the expression of several genes belonging to the pheromone response pathway including *STE5* encoding a scaffold protein that promotes the activation of downstream MAP kinases. Cells lacking Ste5 cannot arrest the cell cycle in response to rapamycin and as a consequence exhibit similar resistance to rapamycin as the H2B R95A mutant. Herein, we show that the H2B R95A mutation weakens the association of H2B with Spt16 a component of the FACT complex (FAcilitates Chromatin Transcription), and an essential factor that interacts with the histone H2A-H2B dimer to promote transcription and preserve chromatin integrity. From a collection of *spt16* mutants, *spt16 E857K* and *spt16-11* showed striking sensitivity to rapamycin as compared to the parent strain. *spt16 E857K* and *spt16-11* expressed distinct forms of Ste5, while a suppressor mutation H2B A84D of the *spt16-11* mutant prevents the expression of Ste5 and confers marked resistance to rapamycin. We interpret these findings to suggest that the Arg95 residue of histone H2B is required to recruit Spt16 to maintain the expression of *STE5*, which performs a role to arrest cells in the G_1_ phase in response to rapamycin.

## Introduction

Rapamycin or its related derivatives such as sirolimus is an immunosuppressant used for treating various diseases that include kidney carcinomas, diabetes, ageing, and neurodegenerative disorders^1–3^. In the budding yeast *Saccharomyces cerevisiae*, rapamycin binds to the peptidyl-prolyl isomerase Fpr1 and this combination inhibits the Target of Rapamycin Complex 1 (TORC1) consisting of either one of the two yeast kinases, Tor1 or Tor2, as well as three additional proteins Kog1, Lst8, and Tco89^4,5^. In response to nutrients, Torc1 controls growth by regulating many macromolecular processes that include cell cycle progression, translation, and transcription^4,6–8^. Exposure to rapamycin leads to the inhibition of Torc1, which causes multiple physiological changes such as alteration in the pattern of gene expression, the phosphorylation status of many factors, cessation of cell growth, degradation of proteins, and finally G_1_ cell cycle arrest^4,9–11^. Despite many years of research, the precise molecular mechanism whereby rapamycin leads to G_1_ arrest in yeast remains unclear. However, it is believed to be the result of a combination of events including the degradation of translational initiation factors^10,12–14^, downregulation of the G_1_ cyclin genes^10^, and stabilization of Sic1 that inhibits the B-type cyclin-dependent protein kinase^15–17^.

We have previously shown that isomerization of the C-terminal domain of the large subunit of RNAPII by the peptidyl-prolyl isomerase Rrd1 plays a prominent role in the redistribution of the polymerase along the genome in response to rapamycin, and this is associated with nucleosomal changes and transcriptional activation implying that histone modifications would govern these processes^6,18–21^. We recently screened an entire collection consisting of 442 histone mutants and reported the identification of nine mutants that affect *S. cerevisiae* response towards rapamycin^22,23^. Eight of the nine histone mutants displayed varying resistance to rapamycin and not to other drugs, while the remaining one mutant H2B E65A exhibited severe sensitivity to rapamycin as well as to other drugs when compared to several other histone mutants in the collection, and with the respective wild-type (WT) background^22,23^.

One of the resistant mutants, in particular, the H2B R95A (arginine substituted for alanine) exhibited remarkable resistance to rapamycin due to its inability to arrest the cell cycle in response to the drug. The H2B R95A mutant lost the ability to express 26 genes of the pheromone response pathway, which is required to mediate α factor-induced G_1_ arrest^23^. *STE5* is a key gene of the pheromone pathway that is drastically downregulated in the H2B R95A mutant^23^. It encodes the scaffold protein Ste5 that performs multiple functions and serves as an essential component for the sequential activation by phosphorylation of the MAPKs, Kss1 and Fus3, required for α factor-induced G_1_ arrest during signalling via the pheromone pathway. Ste5 possesses at least eight phosphorylated sites on the N-terminal, which can be regulated by the G_1_ cyclin Cln2 and its target cell cycle-dependent kinase Cdc28 through a Cln2 docking site located on Ste5^24^. Ste5 can also autoregulate the levels of Cln2^24^. We have shown that cells lacking Ste5 display nearly identical resistance as the H2B R95A mutant towards rapamycin, suggesting that Ste5 is a critical target that responds to the effects of the drug^23^. However, unlike the Ste5 response to α-factor by sequentially phosphorylating the MAPKs, this phenomenon does not appear to occur following rapamycin exposure^23^. In fact, there is a sharp induction of the G_1_ cyclin Cln2 (~ 3- to 4-fold) in the *ste5Δ* mutant within 30 min of exposure to rapamycin as compared to the parent where the induction process is delayed, raising the possibility that rapamycin signalling via the TORC1 is a complex process that involves an interplay with Ste5 leading to cell cycle arrest in the G_1_ phase^23^.

We set out to gain insights on how H2B R95A might regulate the expression of the *STE5* target gene. We exclude the possibility that the regulation would involve epigenetic modification(s) such as methylation on H2B arginine 95 since mass spectrometry data from our studies and that of another group revealed that there is no alteration on this residue in particular towards rapamycin response^23,25^. Herein, we conducted immunoprecipitation analysis coupled with mass spectrometry to search for unique proteins that would interact with FLAG-tagged H2B from the parent, but differ from the H2B R95A mutant. The analysis revealed the Spt16 protein, a component of the FACT complex involved in nucleosomal disassembly and reassembly, interacted weakly with the FLAG-tagged H2B R95A mutant as compared to the H2B WT. Reciprocal immunoprecipitation analysis revealed that a GFP tagged version of Spt16, Spt16-GFP, pulled down significantly less of the FLAG-tagged H2B R95A mutant as compared to the FLAG-H2B WT, indicating that the arginine 95 residue of H2B may play a role in recruiting Spt16. We showed that two variant forms of Spt16, *spt16 E857K* and *spt16-11*, exhibited sensitivity to rapamycin. Interestingly, these two *spt16* mutants caused the expression of abnormal forms of Ste5-GFP. While the *spt16 E857K* mutant weakly expressed a lower molecular weight Ste5-GFP as compared to the parent, the *spt16-11* expressed a normal level of Ste5-GFP, but of a higher molecular weight form. Our data indicate that defects in the Spt16 function can produce altered forms of Ste5 that are unable to mediate normal parental resistance to rapamycin.

## Materials and methods

### Yeast strains, growth media, plasmid, and drugs

The yeast strains and the isogenic mutants used in this work are listed in the Supplementary Table S1^22,23^. The wild-type WT-1 strain and the indicated isogenic mutants were derived from S288C, and the wild-type WT-2 and the isogenic mutants were derived from W303^23,26,27^. Epitope-tagging of strains at the endogenous gene locus was performed as previously described^28^. Cells were grown at 30 °C for 24 h in either Yeast Peptone Dextrose (YPD, FORMEDIUM CCM0105) or SD minimal media. The single-copy plasmid pSTE5-GFP carrying the entire *STE5* gene under its promoter and tagged with GFP was kindly provided by Dr. Peter M. Pryciak (University of Massachusetts medical school, Worcester, MA, USA). All chemical reagents including rapamycin were purchased from Sigma, St Louis, USA.

### Silver staining

SDS gels were stained with the SilverQuest Staining kit according to the manufacturer (Life Technologies).

### Mass spectrometry

Polypeptide bands were excised from the silver-stained gels and subjected to micro-capillary LC/MS/MS analysis mass spectrometry for identification and or modifications (Taplin Mass Spectrometry Facility, Harvard Medical School, Boston, MA, USA).

### RADAR assay

This assay^29^ was adapted for studies with yeast cells. Cells were grown in 1 ml of YPD media and incubated at 30 °C overnight. The next day, the cells were sub-cultured for 2–3 h. An aliquot of 100 µl of the sub-cultured cells was pelleted and resuspended in 150 µl of 100 mM PIPES/KOH pH 9.4 containing 10 mM of DTT. The cells were incubated for 10 min at 30 °C, pelleted and resuspended in 250 µl of YPD containing 0.6 M sorbitol, 25 mM Tris–HCl pH 7.5 and 50 µl of lyticase (5 mg/ml). The cells were incubated for 30 min at 30 °C, then washed twice with 150 µl of YPD containing 0.6 M sorbitol and 25 mM of Tris–HCl pH 7.5. To the cell pellets, 250 µl of MB (4 M guanine thiocyanate, 10 mM Tris–HCL pH 6.5, 20 mM EDTA, 4% Triton X100, 1% Sarkosyl (Sodium lauroyl sarcosinate), and 1% dithiothreitol) and 125 µl of ethanol 100% was added, then stored at − 20 °C for 5 min, centrifuged for 15 min at max speed and the recovered pellet containing the DNA and bound proteins was washed twice with 200 µl of EtOH 75%, each time with centrifugation at max speed for 10 min. The protein-bound DNA pellet was resuspended in 200 µl 8 mM of NaOH and an aliquot of 100 µl was diluted in 200 µl of TBS (50 mM Tris–HCl pH 7.5 and 150 mM NaCl). A sample of 10 µl was used for DNA quantification using Syber Green I Dye and different amounts of the DNA was loaded onto a slot blot equipped with nitrocellulose membrane. Empty wells were loaded with TBS containing bromophenol blue and a gentle vacuum was applied to the slot blot apparatus and once the wells were empty, each well was washed once with 200 µl of TBS and the recovered membrane was cut and each piece was processed by Western blot analysis with the appropriate antibodies^29^.

### Spot test analysis

The strains were grown in YPD broth media at 30 °C for 24 h then the OD 600 nm was adjusted to 1.0. The adjusted OD was serially diluted to 1:10, 1:50, 1:100, 1:500, and 1:1000 in a 96-well plate. Four microliters of each dilution were inoculated on YPD agar without and with 2 ng/ml and 2.5 ng/ml of rapamycin. The plates were incubated at 30 °C for 48 h and (Image Lab Touch Software, BioRad) was used to document the results.

### Co-immunoprecipitation

Cell extracts from H2B WT and H2B R95A were extracted in yeast suspension buffer (YSB) supplemented with protease inhibitors. Protein extracts were incubated with anti-GFP antibodies (Roche Antibodies) at 4 °C overnight and then the immune complex was conjugated to pierce protein A magnetic beads (ThermoFisher Scientific) for 2 h at room temperature. After IP, the beads were washed 4 times thoroughly with TRIS buffered saline containing 0.01% Tween 20 and protease inhibitors. Immunoprecipitated proteins were eluted using 2 × SDS loading buffer and then boiled at 95 °C for 5 min. Denatured proteins were subsequently separated on SDS PAGE gels and immunoblotted against anti-FLAG antibodies (Sigma-Aldrich). For SPT16-GFP tagged strains, cell extracts were incubated with Chromotek-GFP trap magnetic agarose beads (Proteintech) for 2 h at 4 °C. after IP, the beads were washed three times with the wash buffer (10 mM Tris/Cl pH 7.5, 150 mM NaCl, 0.05% Nonidet™ P40 Substitute, 0.5 mM EDTA) supplemented with protease inhibitors. Proteins were eluted in 2 × SDS loading buffer and then boiled at 95 °C for 5 min. Samples were loaded on SDS PAGE gels and blotted against anti-FLAG monoclonal antibody (DYKDDDDK tag Monoclonal antibody (FG4R), MA1-91878, Invitrogen, USA).

### Immunoblot

Exponentially growing yeast strains were pelleted at 3220×*g* for 3 min. Pellets were washed once with sterile water and then resuspended in 20% trichloroacetic acid (TCA). Pellets were transferred to 1.5 ml tubes containing equal volumes of yeast cells extraction glass beads (0.5 mm diameter, BioSpec Cat. No. 11079105). Cells were lysed using a bead mill homogenizer (BeadMill 4, FisherScientific) at 5 m/s for 5 s and repeated 10 times. Supernatants containing protein extracts were collected in fresh tubes. Beads were topped with 150 µl of 5% TCA and subjected to the bead mill homogenizer at 5 m/s for 5 s, 10 times. Supernatants were collected and combined with the first extracts then centrifuged for 10 min at 10,000 rpm at 4 °C. The supernatants were discarded, and existing pellets were washed once in 70% ethanol to remove residual TCA. The pellets were resuspended in 100 µl of 1 × SDS loading buffer. PH was adjusted by adding 5 µl of 1 M Tris base solution. Proteins were then separated on SDS PAGE gels then transferred onto 0.22 µm nitrocellulose membranes. Membranes were immunoblotted against primary antibodies overnight at 4 °C. Antibodies used are anti-GFP IgG (Roche Antibodies), anti-Phospho-p44/42 MAPK (Erk1/2) Thr202/Tyr204 (rabbit mAB 4370, Cell Signaling) generously provided by Dr. Essam Abdelalim (Qatar Biomedical Research Institute, Qatar). Membranes were visualized using Pierce™ ECL Western Blotting Substrate (ThermoFisher Scientific).

### RNA extraction and RT-PCR

Yeast overnight cultures were collected the following day by centrifugation at 4000 rpm for 5 min. The pellets were washed once with sterile water, before proceeding with the RNA extraction protocol using RiboPure™ yeast RNA purification kit (Qiagen) as per the supplier protocol. RNA concentration and purity were measured by Nanodrop 2000. cDNA was prepared from the total RNA (0.5 µg) using high-capacity cDNA reverse transcription kit (ThermoFisher Scientific) according to the recommended protocol. Real-time PCR (qPCR) was performed using SYBR Green Master Mix (ThermoFisher Scientific). Samples were run on Quanti Studio 6 Flex qPCR machine. ACT1 was used as a loading control. Primers used in this study were as follows: ACT1-F, 5ʹ-TGGGTATCCAAGCACATCAA; ACT1-R, 5ʹ-TGATAAACCCGCTGAA CACA; STE5-F, 5ʹ-CGTCCGGAGCAAACTCTATC; and STE5-R, 5ʹ-ATGACCTTAACAGC GGCAAC.

### Software

The quantification analysis was conducted using ImageJ software.

## Results

### H2B R95A weakly interacts with a high molecular weight polypeptide identified as Spt16 belonging to the FACT complex

**Figure 1:**
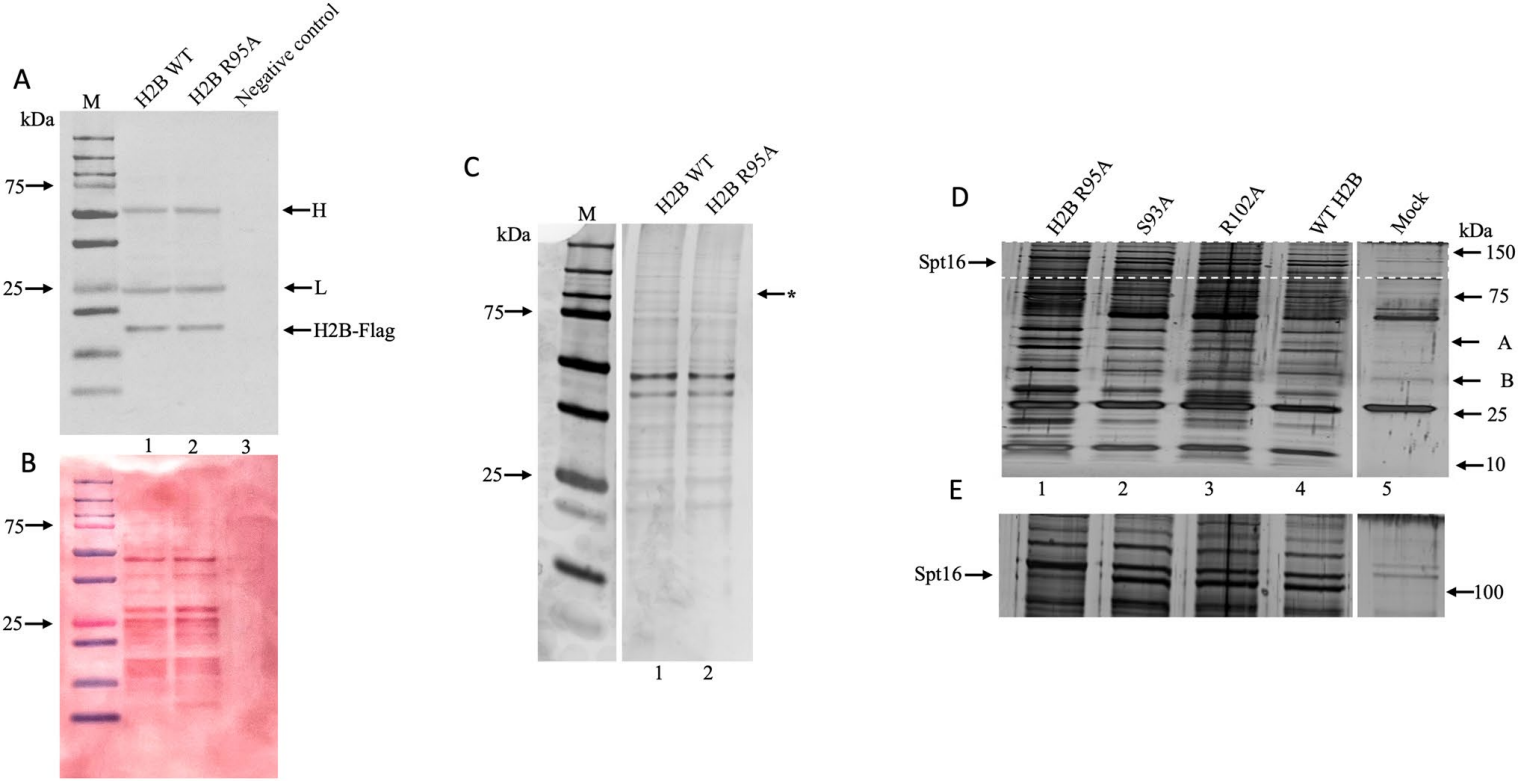
H2B R95A is defective in pulling down a high molecular weight polypeptide identified as Spt16. Total cell extracts were prepared from the H2B WT and H2B R95A strains carrying FLAG-tagged H2B and used in pulldown assays with anti-FLAG magnetic protein A beads. (**A**) Immunoblot analysis of the washed anti-FLAG beads with bound proteins from the H2B WT and H2B R95A mutant strain probed with anti-FLAG antibodies. Negative control, WT strain without FLAG-tagged H2B. The closed arrow shows the pulled-down H2B-FLAG, and opened arrows the heavy and light chains of the anti-FLAG antibodies. (**B**) Ponceau staining of the immunoblot membrane from panel (**A**) showing multiple proteins pulled down from the H2B WT-FLAG and H2B R95A-FLAG extracts with the anti-FLAG beads. (**C**) Silver stained SDS-PAGE analysis of immunoprecipitated H2B-FLAG and associated proteins from the H2B WT-FLAG and H2B R95A-FLAG strains. Total extracts from the indicated strains were pulled down with the anti-FLAG beads, washed and analyzed by SDS-PAGE followed by staining with silver. The asterisk indicates the position of a polypeptide with reduced silver staining intensity. (**D**) Comparison of the proteins pulled down by anti-FLAG beads from the indicated strains and assessed by silver stained SDS PAGE. The experiment was performed as in panel (**C**). The mock pulldown was performed with a WT strain without FLAG-tagged H2B and showing the heavy and light chains of the anti-FLAG antibodies. (**E**) The demarcated area in the upper panel (**D**) is expanded to highlight the 120 kDa protein with reduced staining intensity in the H2B R95A mutant strain. Arrow indicates the position of a polypeptide that was excised from the WT strain and identified by mass spectrometry as Spt16.

Since the H2B R95A variant affects the expression of several genes including the pheromone pathway genes^23^, we examined whether this could be reflected by the proteins differentially associated with H2B R95A as compared to the H2B WT. In the histone mutant collection used in our recent study, the H2B WT and its variants all carried a FLAG epitope tag useful for pulldown assays^23^. Total protein extracts were prepared from exponentially growing cells and the H2B WT-FLAG and H2B R95A-FLAG were pulled down from equal amounts of total protein extracts using protein A beads carrying anti-FLAG antibodies followed by immunoblotting probed with anti-FLAG. Both the H2B WT and H2B R95A were equally pulled down with the anti-FLAG beads (Fig. 1A, closed arrow). The two other bands indicated by the arrows (H and L) showed the position of the heavy and light chains of the FLAG antibodies (Fig. 1A). Ponceau staining showed several proteins were pulled down from H2B WT-FLAG and H2B R95A-FLAG by the anti-FLAG beads, but not in the case of protein extracts derived from a WT strain that lacked FLAG-tagged H2B (negative control) (Fig. 1B, lane 3). The appearance of similar polypeptide bands between the WT and the R95A mutant strain from the pulldown analysis, suggests that the H2B R95A mutation did not prevent it from interacting with proteins (Fig. 1B, lane 2 vs. 1). The proteins associated with the pulldown anti-FLAG beads from the H2B WT and the H2B R95A FLAG extracts were analyzed by SDS-PAGE and stained by silver to closely examine for differences in polypeptide bands. As shown in Fig. 1C, a single high molecular weight polypeptide of ~ 110 kDa (indicated by an asterisk) was weakly detected by silver staining in the pulldown extract derived from the H2B R95A-FLAG strain as compared to the H2B WT (lane 2 vs. 1). Since in this analysis the polypeptide band shown by an asterisk was weak, we repeated the experiment to pulldown substantially more proteins to be stained by silver. In addition, we included two additional H2B mutant strains, H2B S93A-FLAG and H2B R102A-FLAG that carry H2B mutations within the vicinity of the mutant H2B R95A-FLAG. Unlike the H2B R95A variant, neither H2B S93A nor H2B R102A showed resistance to rapamycin and were considered relevant controls^23^. The pulldown followed by silver-stained SDS-PAGE analysis also revealed that a distinct high molecular weight polypeptide band (indicated by the arrow) was sharply reduced from the H2B R95A-FLAG strain, as compared to the H2B WT-FLAG (Fig. 1D, lane 1 vs. 4). This polypeptide was present in the same amount in the pulldown from the two other mutants, H2B S93A-FLAG and H2B R102A-FLAG, as in the case of the H2B WT-FLAG, suggesting that the reduced detection of the high molecular weight polypeptide could be specific to the H2B R95A mutation (Fig. 1D and the perforated region magnified in Fig. 1E). The mock sample was derived from the WT strain where the H2B was not tagged with FLAG and showing the heavy and light chains of the FLAG antibody (Fig. 1D,E, lane 5). Thus, it appears that the H2B R95A mutation hindered the pulldown of the high molecular weight polypeptide band. It is noteworthy that the above pulldown assay with extracts from the H2B S93A and H2B R102A variant also identified unique polypeptides with altered interaction as indicated by arrows A and B, respectively (Fig. 1D, lane 2 and 3), validating this approach that each H2B mutation could affect the recruitment of specific protein(s).

We next excised the ~ 110 kDa polypeptide band from the lane (Fig. 1D, lane 4) with the pulldown sample derived from the H2B WT strain, which corresponded to the high molecular weight polypeptide in the H2B R95A sample with reduced silver staining intensity (Fig. 1D, lane 1). The polypeptide was subjected to mass spectrometry (LC–MS/MS), which revealed that from a total of 20 peptides 17 were derived from a protein identified as Spt16. Spt16 belongs to the FACT complex, a conserved histone-binding heterodimer consisting of Spt16 and Pob3, which plays multiple roles including a chaperone function that is involved in the disassembly and reassembly of the histone H2A-H2B dimer during transcription and replication^30,31^.

### Spt16-GFP efficiently pulldown H2B WT-FLAG and not H2B R95A-FLAG

**Figure 2:**
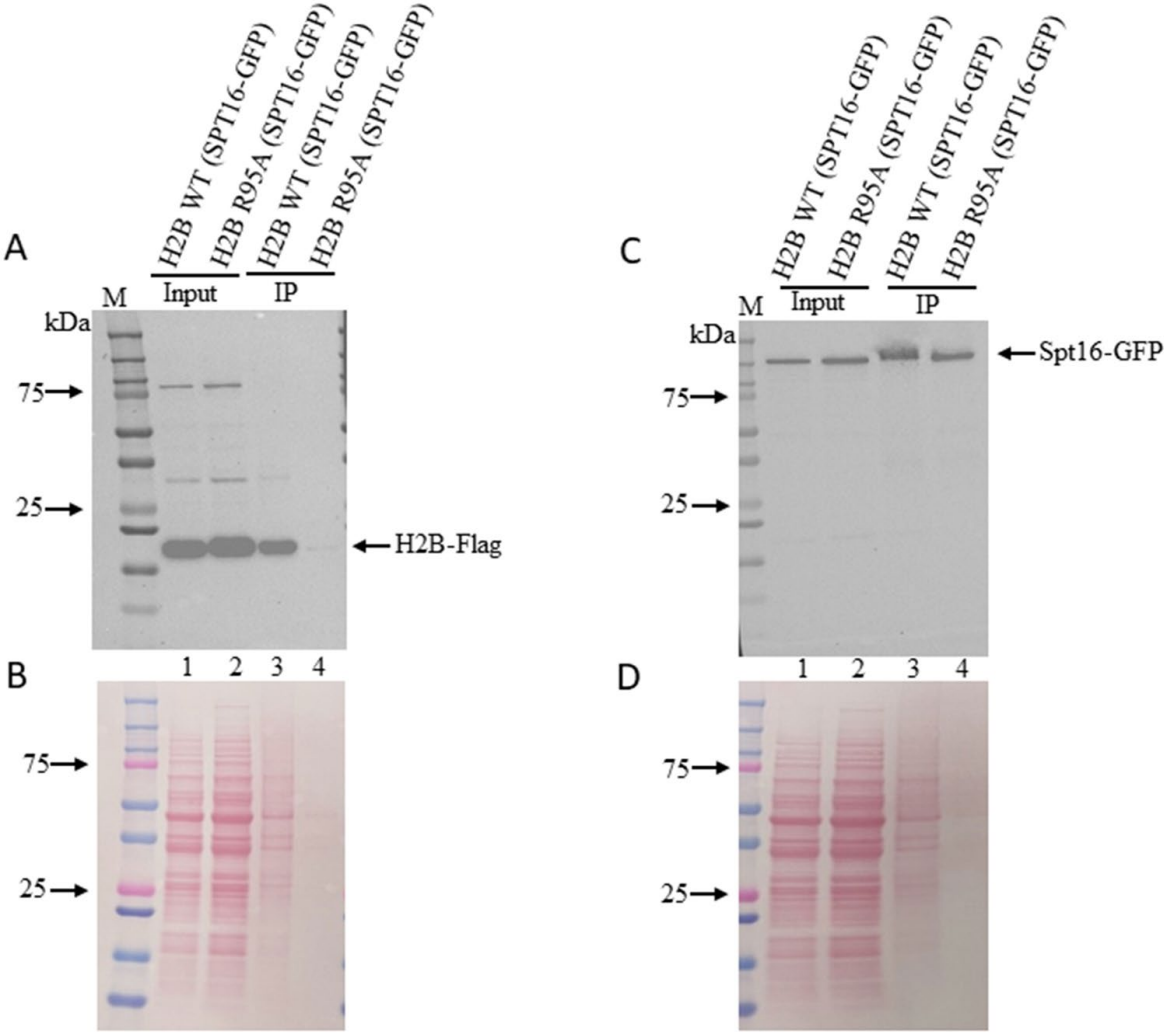
Spt16-GFP efficiently pulls down H2B WT-FLAG and not H2B R95A-FLAG. H2B WT-FLAG and H2B R95A-FLAG cells were tagged at the endogenous locus of *SPT16* with GFP and the total extracts were subjected to immunoprecipitation with anti-GFP beads followed by immunoblot analysis with either anti-FLAG or anti-GFP antibodies. (**A**) Immunoblot detection of H2B-FLAG in the total extracts (input) and bound to the anti-GFP beads (IP). Lanes 1 and 2, total extracts from H2B WT-FLAG and H2B R95A-FLAG cells expressing Spt16-GFP, respectively, and probed with the anti-FLAG monoclonal antibody (FG4R, Invitrogen see “Materials and methods”). Lanes 3 and 4, total extracts from H2B WT-FLAG and H2B R95A-FLAG cells expressing Spt16-GFP, respectively, were subjected to pulldown with anti-GFP beads and probed for the bound fraction of H2B-FLAG. (**B**) The immunoblot of panel (**A**) was stained with ponceau to monitor for equal protein loading of the input. (**C**) Immunoblot detection of Spt16-GFP in the total extracts (input) and bound to the anti-GFP beads (IP). Lanes 1 and 2, total extracts from H2B WT-FLAG and H2B R95A-FLAG cells expressing Spt16-GFP, respectively, and probed with anti-GFP. Lanes 3 and 4, total extracts from H2B WT-FLAG and H2B R95A-FLAG cells expressing Spt16-GFP subjected to pulldown with anti-GFP beads and showing the bound Spt16-GFP. (**D**) The immunoblot of panel (**C**) was stained with ponceau to monitor for equal protein loading of the input.

To validate the above observation, we checked if Spt16 would pulldown H2B. For this experiment, we tagged the *SPT16* gene at the endogenous locus with GFP in the H2B WT-FLAG and H2B R95A-FLAG strains and examined whether anti-GFP beads can pulldown the H2B-FLAG protein from total cell extracts. When equal amounts of extracts from the H2B WT-FLAG and H2B R95A-FLAG strains were probed with an anti-FLAG monoclonal antibody, both strains showed the same amount of H2B-FLAG (Fig. 2A, lanes 1 and 2; and Fig. 2B for ponceau staining). It is noteworthy that the anti-FLAG monoclonal used for Fig. 2A was from a different source than that used for Fig. 1A and it recognized two non-specific bands (Fig. 2A, lane 1 or 2). When equal amounts of extracts from the H2B WT-FLAG and H2B R95A-FLAG strains were also probed with anti-GFP antibodies, both strains showed the same amount of expressed Spt16-GFP (Fig. 2C, lanes 1 and 2; and Fig. 2D for ponceau staining). Thus, the R95A mutation did not interfere with the stability of either the H2B-FLAG or the Spt16-GFP as compared to the parent.

Immunoprecipitation analysis with the anti-GFP beads revealed that Spt16-GFP efficiently pulled down the H2B WT-FLAG, and only weakly the H2B R95A-FLAG, as detected by immunoblot probed with anti-FLAG (Fig. 2A, lanes 3 and 4, respectively). In these experiments, the anti-GFP beads pulled down the same amount of Spt16-GFP from both the H2B WT-FLAG and H2B R95A-FLAG strains, as detected by immunoblot with anti-GFP (Fig. 2C, lanes 3 and 4, respectively, and an independent pulled down of Spt16-GFP is shown in Supplementary Fig. S1, excluding the possibility that the weak pulled down of H2B R95A-FLAG by Spt16-GFP is due to Spt16-GFP differential binding to the anti-GFP beads). Collectively, these data indicate that Spt16 is weakly interacting with the H2B R95A mutant.

In a separate experiment, we used the RADAR assay that monitors for chromatin-bound proteins^29^. The assay revealed that Spt16-GFP was weakly attached to chromatin derived from the H2B R95A mutant as compared to WT when the same amount of total DNA was analyzed (see Supplementary Fig. S2, lane 2 vs. 1). A control DNA binding protein, Apn1, showed a similar association with the chromatin derived from either the WT H2B or the H2B R95A mutant strain (Fig. S2). Consistent with these findings, several studies demonstrated a physical interaction between H2B and Spt16^32,33^. Thus, it would appear that the Arg95 residue of H2B is indeed required for the efficient binding of Spt16 to chromatin.

### Variants of Spt16 display sensitivity to rapamycin

**Figure 3:**
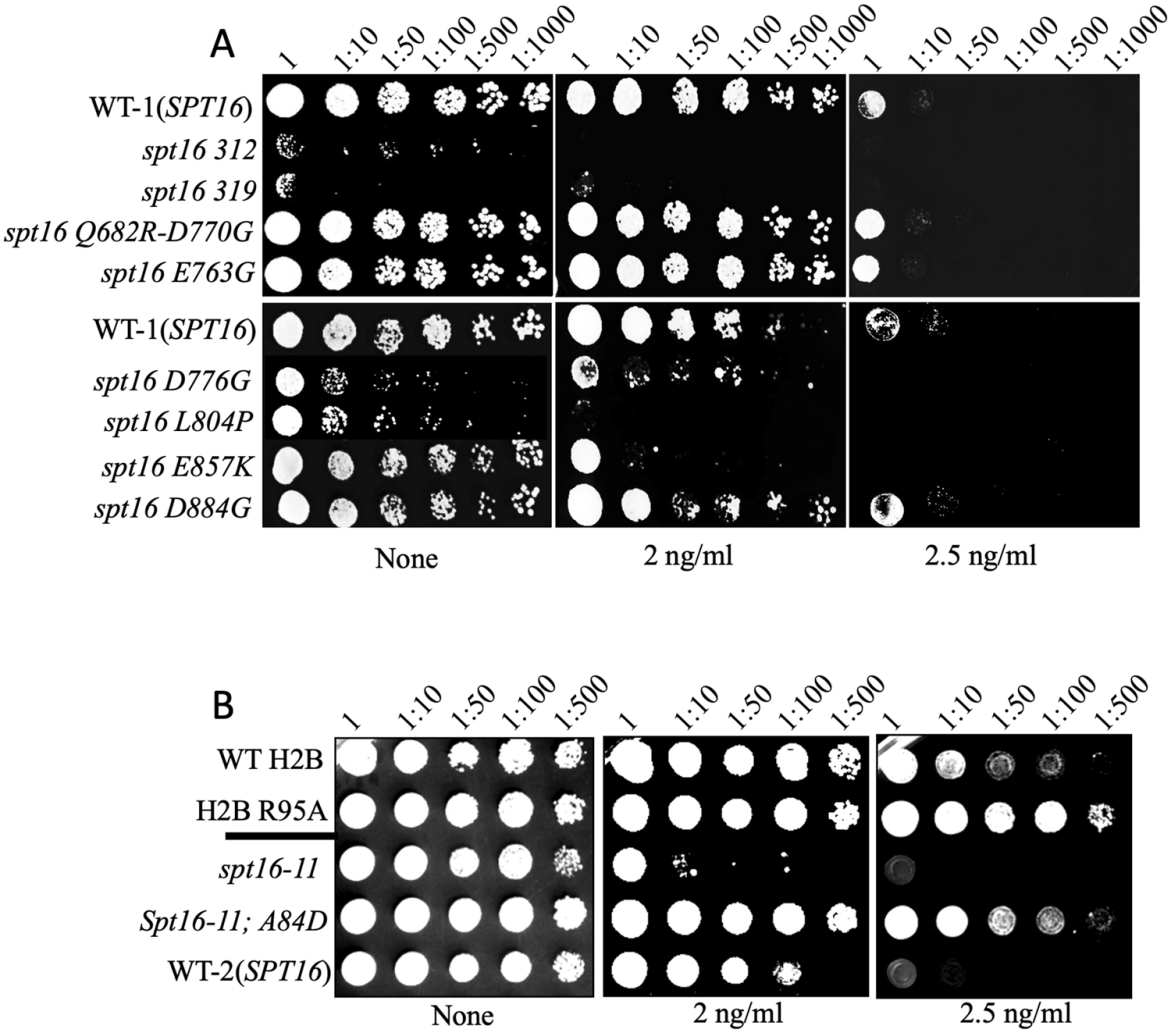
Spot test analysis showing the sensitivity and resistance of spt16 mutants towards rapamycin. (**A**) The indicated WT and isogenic *spt16* mutants were grown overnight and next day serially diluted and spotted onto plates without and with rapamycin. (**B**) The spot test was performed as in panel (**A**). The plates were photographed 48 h following incubation at 30 °C.

Stevens et al., reported the isolation of several* spt16* mutants that are defective in transcription-linked nucleosome reassembly while maintaining the essential functions of Spt16^32^. In the *spt16* mutant collection, some interact weakly with histone H2B raising the possibility that they may be defective in response to rapamycin^33^. Since H2B R95A is defective in transcription and displays striking resistance towards rapamycin, we tested whether any of the *spt16* mutants would exhibit a rapamycin phenotype using spot test analysis. Amongst these mutants, none showed more than WT resistance to rapamycin, and some grew poorly such as *spt16-312*, the triple mutant *spt16-319* (*L804P L946S E1004G*), and *spt16-D776G*, thus their responses towards rapamycin cannot be properly assessed as compared to the isogenic parental strain WT-1(*SPT16*), derived from the S288C genetic background (Fig. 3A, see Table S1). In contrast, *spt16 E857K* grew normally as the WT-1, but exhibited mark sensitivity to rapamycin (Fig. 3A and Supplementary Fig. S3). Since the *spt16 E857K* mutation is unable to properly bind to H2A-H2B dimer and reestablish the repressive nucleosome following the elongating RNAPII^32^, this mutation may affect the expression of genes required to mount a response to rapamycin.

### The *spt16-11* allele carrying the histone H2B A84D mutation is resistant to rapamycin

An independent study by McCullough et al., 2011, reported several phenotypes associated with another allele, *spt16-11*, of the *SPT16* gene derived from a different genetic background, namely W303^34^. We examined whether this *spt16-11* mutant would display an altered response to rapamycin. Spot test analysis revealed that the *spt16-11* allele was sensitive to rapamycin, as compared to the parental strain W303 (WT-2) (Fig. 3B). The study by McCullough also isolated several histone mutations, including H2B A84D that strongly suppressed the phenotypes of the *spt16-11* mutation^34^. In addition, they showed by in vitro analysis that the H2B A84D mutant caused nucleosome instability by enhancing dimer displacement, thus weakening the histone dimer: tetramer interface, a function attributed to the FACT complex^34^. Thus, the H2B A84D mutation may not be able to maintain the nucleosome structure and the repressive chromatin, thus leading to alter gene expression patterns^34^. Interestingly, the sensitivity of the *spt16-11* mutant to rapamycin was completely abolished upon the introduction of the H2B A84D mutation into the strain to create a double mutation *spt16-11*; H2B A84D (Fig. 3B). This double mutant conferred striking resistance to rapamycin when compared to the parent WT-2, and similar to the H2B R95A mutant (Fig. 3B). Taken together, the data indicate that Spt16 plays a role in rapamycin response and this involves cooperation with histone H2B.

### Spt16 mutants express different forms and levels of Ste5-GFP

**Figure 4:**
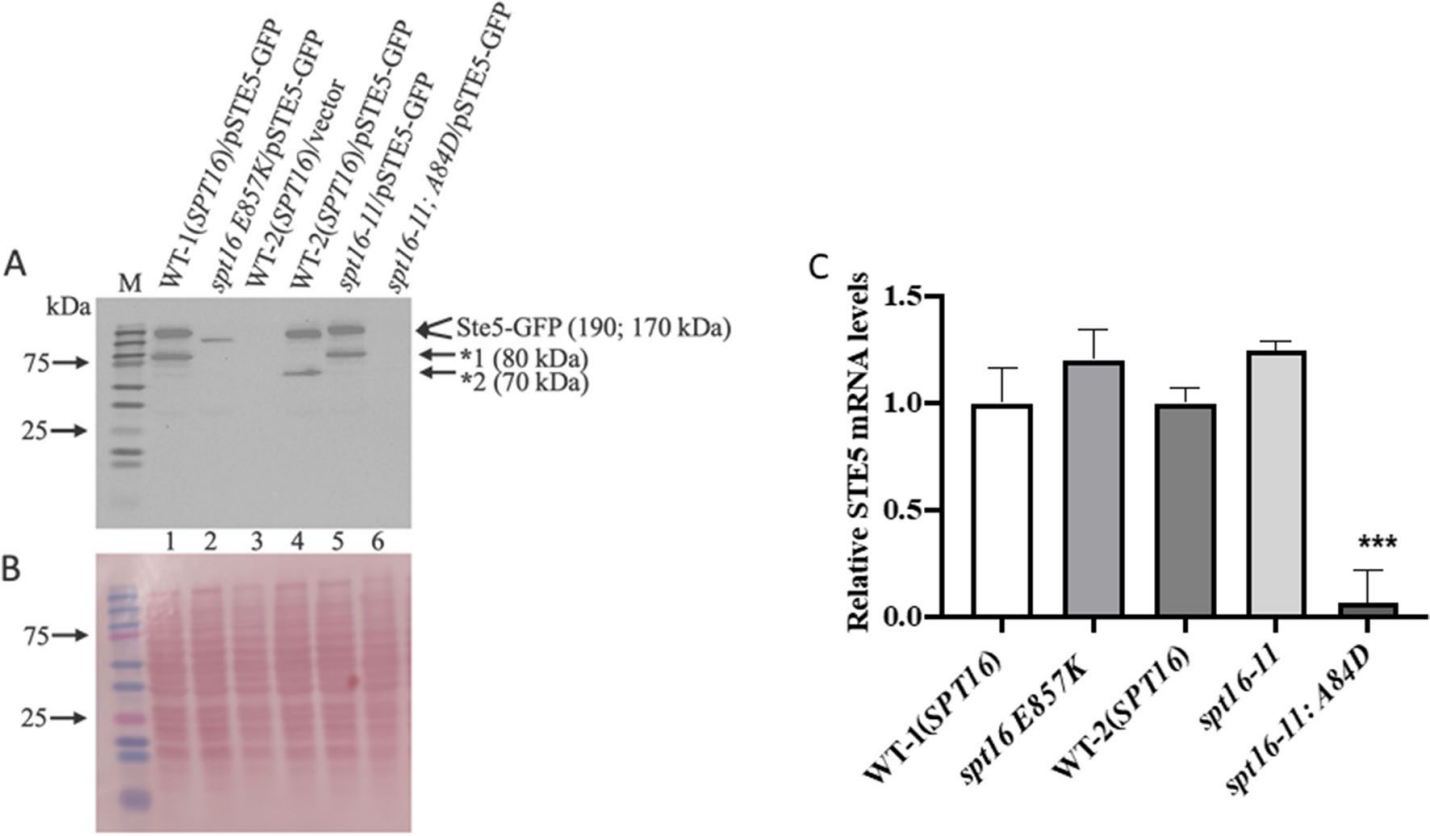
Spt16 mutants expressed different forms and levels of Ste5-GFP. (**A**) Immunoblot analysis of Ste5-GFP expression level in the indicated wild type (WT) and *spt16* mutants. The plasmid pSTE5-GFP or empty vector was introduced into the WT-1 (derived from the parent S288C) and the isogenic *spt16 E857K* mutant, the WT-2 (derived from the parent W303) and the isogenic *spt16-11* single mutant and the *spt16-11*; A84D double mutant. Total cell extracts were prepared from the strains using trichloroacetic acid (TCA) and processed for immunoblot analysis probed with anti-GFP antibodies. (**B**) Ponceau staining to monitor for protein loading. *M* prestained protein markers in kDa. Arrows indicate the position of the GFP-tagged proteins (~ 190 and 170 kDa), and the asterisks indicate the fragmented sp of Ste5-GFP (~ 80 and 70 kDa). A repeat of this experiment was performed using a gradient gel and the data is shown in Supplementary Fig. S4A,B. (**C**) *STE5* mRNA expression levels by RT-qPCR in WT-1(*SPT16*), *spt16 E857K*, WT-2 (*SPT16*), *spt16-11* and *spt16-11; A84D* strains. *ACT1* was used as a loading control.

We recently showed that the expression of many genes belonging to the pheromone pathway including *STE5* is drastically downregulated in the rapamycin-resistant H2B R95A mutant^23^. Ste5 is a scaffold protein that promotes the sequential phosphorylation of the kinases Ste11, Ste7, Fus3, and Kss1, which culminated with the MAPKs Kss1 and Fus3 being activated^35–37^. The activated Fus3 leads to the downregulation of the G_1_ cyclins including Cln1 and Cln2 causing cell cycle arrest^38,39^. We checked whether the Ste5 expression level would be affected by variants of Spt16 that have defects in interacting with the H2A-H2B dimer^32,34^. To assess this, the single-copy plasmid pSTE5-GFP expressing Ste5 from its promoter and as a GFP fusion protein was introduced into the rapamycin-sensitive *spt16* mutants, *spt16 E857K* and *spt16-11*, and the rapamycin-resistant double mutant *spt16-11;* H2B A84D, as well as the respective WT strains. Following TCA extraction, the level of expression of the Ste5-GFP protein in the above strains was determined using an anti-GFP antibody. Immunoblot analysis revealed that Ste5-GFP was expressed at the expected size of the Ste5-GFP (~ 190 kDa) in the parent strain WT-1 (Fig. 4A, lane 1). This parent strain also expressed a lower molecular weight specie of Ste5-GFP (~ 80 kDa) that could be a proteolytic fragment (Fig. 4, lane 1). The rapamycin-sensitive mutant strain, *spt16 E857K*, did not express the full-length Ste5-GFP, but weakly expressed a lower molecular weight form (~ 140 kDa) and no other detectable specie, as compared to the parent strain WT-1 (Fig. 4A, lane 2 vs. 1, and Fig. 4B for ponceau staining; similar data were obtained in an independent experiment as shown in Supplementary Fig. S4A, except the samples were processed on a gradient gel). Although the rapamycin-sensitive *spt16* mutant, *spt16 E857K,* showed a lower level of Ste5-GFP, it expressed a normal level of the *STE5* gene as determined by qPCR (Fig. 4C). These findings suggest that the expression of the full-length form of Ste5 depends upon the native Spt16 protein.

In contrast, the mutant strain *spt16-11* also exhibiting rapamycin sensitivity expressed a significantly higher form of Ste5-GFP (~ 190 kDa) as compared to its parent (~ 170 kDa) strain WT-2 (Fig. 4A, lane 5 vs. 4; Supplementary Fig. S4A showing an independent experiment). Both the *spt16-11* mutant and the WT-2 strain expressed Ste5-GFP to the same level (Fig. 4A, lane 5 vs. 4; Supplementary Fig. S4A) consistent with the expression level of the *STE5* gene (Fig. 4C). Surprisingly, the rapamycin-resistant double mutant *spt16-11; H2B A84D* did not express the full-length Ste5-GFP nor the *STE5* gene (Fig. 4C). This latter observation is reminiscent of our recent findings whereby the histone mutant H2B R95A completely abolished the expression of the *STE5* gene and caused cells to be resistant to rapamycin^23^. Altogether, the data suggest that the altered forms of Ste5-GFP detected in the *spt16* mutants may dampen the signal required to allow some cells to escape the G1 arrest^40^.

### The rapamycin-sensitive *spt16* mutants harbour induced levels of phosphorylated Fus3

**Figure 5:**
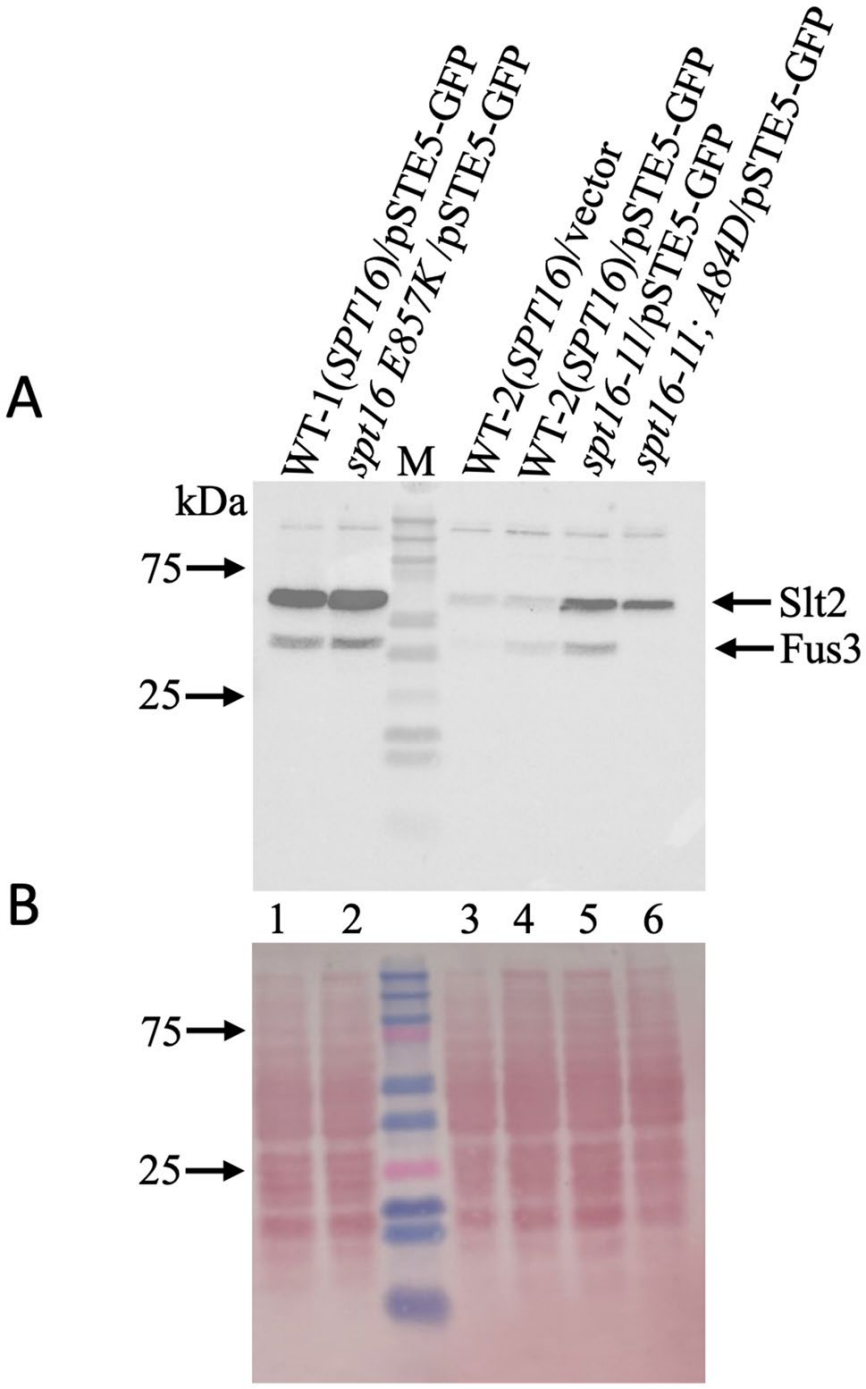
Comparison of the endogenous level of activated Fus3 in the indicated WT and the *spt16* mutant strains. (**A**) Exponentially growing cells from the indicated strains were harvested and subjected to total protein extraction by trichloroacetic acid (TCA). The TCA-extracted proteins were analyzed by immunoblot and probed with anti-Erk1/2 to detect the MAPKs. The antibody detects three known proteins in yeast, the kinases Slt2, Kss1, and Fus3, but Kss1 detection is extremely weak in the S288C and W303 genetic background. (**B**) Ponceau to monitor for protein loading from the TCA samples. *M* pre-stained protein markers in kDa. A repeat of this experiment was performed using a gradient gel and the data is shown in Supplementary Fig. S4C,D.

Functional Ste5-GFP leads to the activation of the downstream MAP kinases such as Fus3 and Kss1 of the pheromone pathway^40^. The activated MAP kinases, Fus3 and Kss1, are phosphorylated and can be detected with the anti-ERK1, 2 antibodies, which also detect another kinase Slt2^23^. We examined if these *spt16* mutants with altered forms and expression levels of Ste5-GFP would compromise the endogenous levels of activated Fus3 and Kss1. The data revealed that *spt16 E857K* and *spt16-11* showed elevated levels of Fus3 when compared to the respective WT strains (Fig. 5, lane 2 vs. 1 and lane 5 vs. 4, respectively; see also Supplementary Fig. S4C for an independent experiment). Unlike Fus3, Kss1 is a weakly expressed MAP kinase and is not readily detected with the anti-ERK1/ERK2 antibodies^23^. Interestingly, the H2B A84D mutation that rescued *spt16-11* phenotypes and prevented the expression of Ste5-GFP blocked the detection of the MAP kinase Fus3. Thus, it would appear that Spt16 plays a role in maintaining the proper expression and molecular form of Ste5 and consequently the associated MAP kinases that control the G_1_ cyclins Cln1 and Cln2.

### The WT and the *spt16-11* mutant show differential levels of Cln2 in response to rapamycin

**Figure 6:**
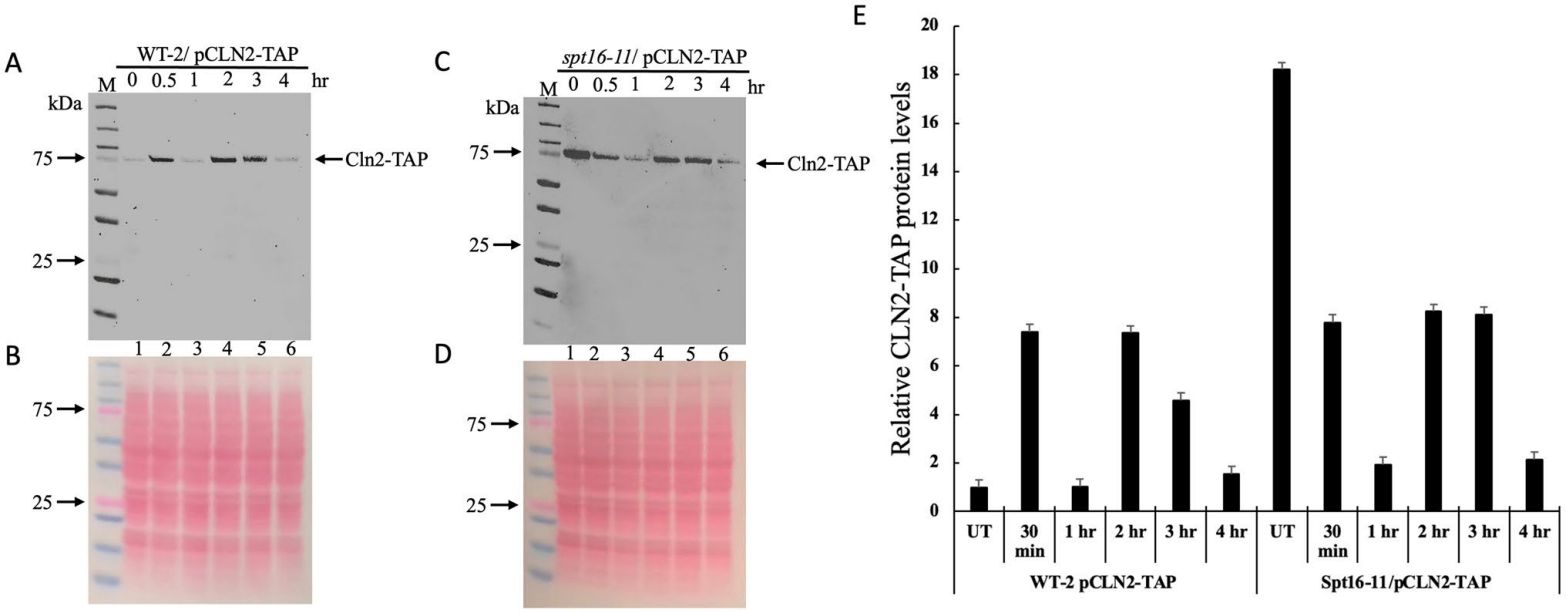
Comparison of Cln2-TAP expression levels in the WT and the *spt16-11* mutant strain in response to rapamycin. (**A**,**C**) Exponentially growing cells from the WT-2 strain and the isogenic *spt16-11* mutant carrying the TAP tag at the endogenous *CLN2* gene locus were treated without (time zero) and with rapamycin (200 ng/ml). Samples were taken at 0.5, 1, 2, 3 and 4 h for total protein extraction by TCA and analyzed by immunoblot probed with anti-TAP, which recognizes the protein A domain of the Cln2-TAP-tagged fusion protein. (**B**,**D**) Ponceau staining to monitor for protein loading from the TCA samples. *M* prestained protein markers in kDa. (**E**) Quantification of Cln2-TAP levels relative to the untreated WT-2 and the untreated isogenic spt16-11 mutant (i.e., at zero time point).

We checked whether the sensitivity of the *spt16-11* mutant to rapamycin could be due to the altered forms of Ste5 affecting Cln2 expression and thus prevent growth in the presence of the drug. To do this, the *CLN2* gene in the *spt16-11* mutant and the isogenic WT was tagged at the endogenous locus with the Tandem Affinity Purification tag that consists of Protein A and Calmodulin binding domain, which we have previously reported for the BY4741 genetic background^23^. The *CLN2* gene could not be tagged in the *spt16 E857K* mutant due to the lack of a selectable marker, and thus, this mutant was omitted from further analysis. For this experiment, the cells were untreated (time 0 h) or treated with rapamycin and cultures harvested at the indicated time (0.5–4 h) and processed for immunoblot analysis probed with a peroxidase-antiperoxidase complex (PAP, Sigma 2026), which detects protein A of the TAP tag. As shown in Fig. 6A, the Cln2-TAP expression level in the WT was induced at least sixfold within 0.5 h upon treatment with rapamycin, as compared to the untreated (lane 2 vs. 1, and Fig. 6B, ponceau staining of the membrane was used to assess for equal loading of the protein samples). The high Cln2-TAP level disappeared in the next 0.5 h and restored to the untreated level within the 1 h treatment (Fig. 6A, lane 3), however, Cln2-TAP was again induced by 2 h under the sustained treatment and disappeared by 4 h (lanes 4–6). Unlike the WT, the *spt16-11* mutant already maintained a very high level of Cln2-TAP in the absence of rapamycin treatment (Fig. 6C, lane 1 vs. Fig. 6A, lane 1). Upon rapamycin treatment of the *spt16-11* mutant, there was no apparent induction of Cln2-TAP at 0.5 h and instead, it substantially decreased (Fig. 6C, lane 2 vs. lane 1). At the 1 h time treatment, the Cln2-TAP was further diminished in the *spt16-11* mutant (Fig. 6C, lane 3), and began to reestablish its expression by 2 h of the sustained treatment followed by its degradation at 4 h (Fig. 6C, lane 4). The data indicate that Cln2 is undergoing a cyclic response, induction followed by its degradation and that its induction is required to establish WT resistance to rapamycin.

## Discussion

In this study, we reported that the arginine 95 residue of histone H2B is required to efficiently interact with the Spt16 protein, a component of the FACT (FAcilitates Chromatin Transcription/Transactions) complex that plays multiple roles including transcription, replication, and chromatin remodelling^27,34,41–43^. The FACT complex possesses two conserved and essential subunits, Spt16 and Pob3, and serves as a histone chaperone that is required to disassemble the nucleosomal barrier to DNA transactions and allow, for example, transcription elongation by RNA polymerase II. Recent high-resolution genome mapping revealed that in *S. cerevisiae* FACT binds to the transcribed chromatin. It is believed to initiate binding to the first nucleosome that is partially loosened by the engaging RNA pol II followed by a processive mechanism to spread to the downstream nucleosomes^44^. FACT bound to the transcribing chromatin promotes the disassembly of the nucleosomes in front of the moving transcription complex, and then the reassembly of the nucleosomes behind the transcriptional machinery^27,44^. This re-establishment of the nucleosomes recreates the repressive state to prevent unwanted transcription such as transcription from a cryptic promoter, which is embedded in some genes and can initiate transcription^45–47^. It is predicted that at least 15% of yeast genes carry cryptic promoters and these are repressed by the normal function of the FACT complex^48^.

Herein, we provide evidence that the substitution of Arg95 with alanine in histone H2B weakened the interaction with Spt16, and thus this would likely impede FACT function. Other H2B mutants have been previously reported that interrupted FACT binding to the H2A-H2B dimer and these include amino acid substitution at the essential histone residues H2B L109 and H2B M62 and the non-essential residue H2B E57^49,50^. In the latter case, mutating the glutamic acid residue H2B E57 to alanine diminished its interaction with Spt16^50^. While the H2B E57A mutant shared phenotypes in common with *spt16* mutants, such as sensitivity to hydroxyurea and a propensity to engage in cryptic transcription, this mutant exhibited parental response towards rapamycin^50^. However, we have not yet examined if this particular mutant is expressing an altered molecular weight form of Ste5-GFP. Thus, our recent report demonstrating that the H2B R95A mutant is unable to express several genes, including genes of the pheromone response pathway, might be explained by its inability to properly recruit Spt16 which ultimately affects the FACT complex to promote gene expression^23^. In our experiments, we did not examine whether the Pob3 component of the FACT complex was also weakly pulled down by H2B R95A-FLAG. Since both Spt16 and Pob3 are in a complex that interacts with the H2A-H2B dimer, we anticipate that Pob3 would also be weakly pulled down by H2B R95A-FLAG. However, we cannot exclude the possibility that the native H2B R95 may only recruit Spt16, and not Pob3, as highlighted by recent studies showing that Spt16 can act independently of the FACT complex to modulate transcription^51,52^. It would be interesting to explore in the future whether yeast mutants, such as *san1Δ*, with the ability to maintain higher levels of Spt16 while keeping the Pob3 level constant can activate *STE5* gene expression^51^.

Because the H2B R95A mutant sharply diminishes the expression of *STE5*, and both H2B R95A and the *ste5* mutants displayed nearly similar hyperresistance phenotype to rapamycin prompted us to check whether *spt16* mutants would engender similar resistance to rapamycin and thus interfere with the expression of *STE5*^23^. From a small collection of *spt16* mutants, we found two mutants, *spt16 E857K* and *spt16-11*, that showed sensitivity to rapamycin, but none of the *spt16* mutants alone displayed resistance to the drug. Indeed these two *spt16* mutants were unable to express the native form of Ste5-GFP, in particular, the *spt16 E857K* expressed a lower molecular weight specie and the *spt16-11* expressed a higher molecular weight specie. Although the lower molecular weight Ste5-GFP polypeptide ~ 140 kDa found in the *spt16 E857K* mutant can be explained by proteolytic cleavage of the native Ste5-GFP, the most likely explanation for this polypeptide specie is that transcription starts from a cryptic promoter within the ORF of the *STE5* gene. The *spt16 E857K* substitution mutation was identified through its inability to prevent transcription from a cryptic internal promoter located within the open reading frame of the *FLO8* gene, which is normally repressed by the native nucleosomal structure in the parent *SPT16* strain^26,32,47^. If indeed the Ste5-GFP polypeptide is the result of an internal transcription start site, this shorter protein may be responsible for sensitizing the *spt16 E857K* mutant to rapamycin. The observation that the *spt16 E857K* mutant showed induced activation of the Fus3 MAP kinase as compared to the parent is consistent with a defect in the scaffold function of the shorter Ste5-GFP protein in controlling the activation of Fus3 (Fig. 5). The higher activation of Fus3 would subsequently lead to more efficient suppression of the G1 cyclins such as Cln2 required to allow cells to transit from the G1 to the S phase to promote some growth in response to rapamycin (see below). We have not determined the location of the cryptic promoter within the *STE5* gene that produced the shorter Ste5-GFP polypeptide in the *spt16 E857K* mutant strain, and whether this specie would sensitize the WT strain to rapamycin or the *ste5* null mutant that is known to display striking resistance to the drug^23^.

The *spt16-11* mutant also showed sensitivity to rapamycin and was previously reported to exhibit several phenotypes that affect both transcription and replication^53^. This mutant displays sensitivity to temperature, as well as to hydroxyurea that inhibits ribonucleotide reductase causing depletion of dNTPs and subsequently stalling replication^53^. The *spt16-11* mutation is unable to cause the FACT complex to disrupt the histone H2A-H2B:(H3–H4)2 dimer: tetramer interface to promote transcription^34^. Herein, we observed that the *spt16-11* mutant expressed nearly the same amount of Ste5-GFP as the WT, but the expressed protein displayed a higher molecular weight (Fig. 4). This higher molecular weight form of Ste5-GFP may be responsible for the observed hyperactivated form of Fus3 (Fig. 5), which plays a role in suppressing the expression of the G_1_ cyclins^23^. Unexpectedly, the *spt16-11* mutant maintained substantially higher levels of the Cln2 protein, nearly 15-fold higher than the parent strain (Fig. 6C). This is counter-intuitive to the normal signalling function of the observed activated Fus3, as it would be expected to mediate the repression of the G_1_ cyclins. Based on our findings and the known functions of FACT, it would appear that the *spt16-11* mutation is unable to reestablish the repressive chromatin thereby allowing continuous expression of Cln2.

We have recently shown that rapamycin treatment induced the synthesis of Cln2 within 30 min, and this observation is reproduced in the present work with a different genetic background strain, W303, and the parent of the *spt16-11* mutant^23^. The newly synthesized Cln2 is required to activate Cdc28 to promote G1 to S phase transition which would commit some cells to proliferate in the presence of rapamycin before growth ceases. However, the *spt16-11* mutation failed to induce Cln2 in response to rapamycin and the high levels of preexisting Cln2 in this mutant began to drastically disappear (Fig. 6). We have no clear explanation as to why the *spt16-11* mutant failed to induce Cln2, although this observation is consistent with the mutant showing sensitivity to rapamycin; mutants that are resistant to rapamycin such as *ste5Δ* show rapid induction of Cln2^23^. However, we offer two possible explanations for the failed Cln2 induction by the *spt16-11* mutant. One possibility is that the modified Ste5 in the *spt16-11* mutant may no longer allow docking of Cln2 onto its Leucine-Proline rich sequence blocking Cln2 from maintaining the transcriptional repressor Whi5 in the hyperphosphorylated form and setting Whi5 free from inhibiting the SBF transcription factor that activates, for example, *CLN2* gene expression^54^. Alternatively, the failed Cln2 induction may be a consequence of the *spt16-11* mutation causing a defective FACT complex that is unable to recruit the transcription factors MBF and SBF to the promoter elements on the *CLN2* gene^55^.

We also chose to examine whether the H2B A84D suppressor mutation of the *spt16-11* mutant would restore to this strain parental resistance to rapamycin since this H2B mutation has been shown to correct the hydroxyurea and temperature sensitivities of the *spt16-11* mutant^34^. Surprisingly, the H2B A84D suppressor mutation completely blocked the expression of Ste5-GFP and conferred upon the *spt16-11* mutant striking resistance to rapamycin (Fig. 3B). We reasoned that the simplest explanation for the failed expression of Ste5-GFP in the *spt16-11*; H2B A84D double mutant could be due to the proximity of the negatively charged Ala84Asp substitution relative to the positively charged Arg95 residue, and this would likely interfere with the recruitment of an already defective spt16-11 preventing the expression of the Ste5-GFP protein. It is noteworthy that the H2B A84D mutation alone did not affect rapamycin response or hydroxyurea sensitivity^34^. However, we cannot exclude other possibilities that the *spt16-11*; H2B A84D double mutant may use a downstream cryptic promoter that produces unstable mRNA or the altered nucleosome may facilitate the expression of noncoding miRNAs that block the expression of Ste5-GFP^56^. In support of this latter possibility, the *spt16-11* mutant shared similar phenotypes with another *spt16* mutant, *spt16-G132D*, that exhibits increased cryptic gene transcription and cryptic noncoding transcription associated with nucleosomal changes^56^.

In short, we have provided evidence to explain the defective gene expression caused by the H2B R95A mutant^23^. The H2B Arg95 residue appears to play a vital role in recruiting Spt16, a component of the FACT complex, required to promote the disassembly and reassembly of the nucleosomes to allow efficient gene expression. So far, we and others have not been able to identify any modification on H2B Arg95^23^. As such, it remains unclear how the H2B Arg95 residue performs its role to recruit Spt16 to promote the expression of specific subsets of genes such as genes of the pheromone response pathway and not genes belonging to another physiological pathway.

## Supplementary Information

Supplementary Information.

## Data Availability

The datasets used and/or analysed during the current study are available from the corresponding author upon reasonable request.
