# Supplementary material for: The histone H2B Arg95 residue efficiently recruits the transcription factor Spt16 to mediate Ste5 expression of the pheromone response pathway
Source: Sci Rep. 2023 Jun 22;13:10189. doi: 10.1038/s41598-023-37339-y (PMC10287706; doi:10.1038/s41598-023-37339-y)

The histone H2B Arg95 residue efficiently recruits the transcription factor Spt16 to mediate Ste5 expression of the pheromone response pathway

Abdallah Alhaj Sulaiman[1], Reem Ali[1], and Dindial Ramotar[1, a]

Running title: Arginine 95 of H2B is essential for Spt16 recruitment

[1]Division of Biological and Biomedical Sciences, College of Health and Life Sciences, Hamad Bin Khalifa University, Education City, Qatar Foundation, Doha, Qatar, P.O.Box: 34110

[a] Corresponding author Email: dramotar@hbku.edu.qa

Table S1. Yeast strains and mutants used in this study

**Strains**	**Genotype**	**Source**
BY4741	Parent, *Mat a, his3-1, leu2-0, met15-0, ura3-0*	Euroscarf (Frankfurt, Germany)
ste5Δ::KANMX	isogenic to BY4741, except deleted for the *STE5*	This work (resistant to rapamycin)
ste2Δ::KANMX	isogenic to BY4741, except deleted for the *STE2*	This work
FY406	The H2A/H2B collection is in FY406 MATa (hta1-htb1)Δ::LEU2, (hta2-htb2) Δ::TRP1, his3 Δ 200 leu2 Δ 1 ura3-52 trp1 Δ 63 lys2-128 Δ <pSAB6 (HTA1-HTB1-URA3). The covering plasmid for H2A/H2B uses the His marker.	Ali Shilatifard (Kansas, USA)
YBL574	The H3/H4 collection is in Winston's YBL574 (MATa, leu2Δ1, his3Δ200, ura3-52, trp1Δ63, lys2-128δ, (hht1-hhf1)ΔLEU2 (hht2-hhf2)Δ::HIS3 Ty912Δ35-lacZ::his4, {pDM9-HHT1-HHF1-URA3}. The covering plasmid for H3/H4 uses the Trp marker.	Ali Shilatifard
TAP tag strains collection	MATa his3Δ1 leu2Δ0 met15Δ0 ura3Δ0	Dharmacon
Y1a	Parent W303 (leu2-3, 112, trp1-1, can1-100, ura3-1, ade2-1, his3-11,15)	Lab strain
ste5Δ::LEU2	Isogenic to W303, except deleted for *STE5*	This work (resistant to rapamycin)
WT-1 (*SPT16*)	Strain AFO400 (*MATα ade2Δ ade3Δ his4-912δ lys2-128δ leu2-3,112 trp1-Δ1 ura3-52 spt16Δ::kanMX4* [pRS314-SPT16), a derivative of the S288C was chromosomally deleted for SPT16 but with viability maintained by separate SPT16 plasmid.	Richard A. Singer, Dalhousie University, Halifax, Nova Scotia, Canada
spt16 312	Isogenic to WT-1(*SPT16*), except carrying [pRS314-spt16-312]	Richard A. Singer
spt16 319	Isogenic to WT-1(*SPT16*), except carrying [pRS314-spt16-319]	Richard A. Singer

spt16 Q682R-D770G	Isogenic to WT-1(*SPT16*), except carrying [pRS314-spt16-Q682R-D770G]	Richard A. Singer
spt16 E763G	Isogenic to WT-1(*SPT16*), except carrying [pRS314-spt16-E763G]	Richard A. Singer
spt16 D776G	Isogenic to WT-1(*SPT16*), except carrying [pRS314-spt16-D776G]	Richard A. Singer
spt16 L804P	Isogenic to WT-1(*SPT16*), except carrying [pRS314-spt16-L804P]	Richard A. Singer
spt16 E857K	Isogenic to WT-1(*SPT16*), except carrying [pRS314-spt16-E857K]	Richard A. Singer
spt16 D884G	Isogenic to WT-1(*SPT16*), except carrying [pRS314-spt16-D884G]	Richard A. Singer
WT H2B	FY406	Ali Shilatifard
H2B R95A	Derived from FY406	Ali Shilatifard
WT-2 (*SPT16*)	MATa ura3-Δ0 leu2-Δ0 trp1-Δ2 his3 lys2-128@ HTB1(30, URA3) HTB2(30, His3MX)	Tim Formosa (USA)
spt16-11	MATa ura3 leu2 trp1 his3 lys2-128@ HTB1(30, URA3) HTB2(30, His3MX) spt16-11	Tim Formosa
H2B A84D	MATa ura3-Δ0 leu2-Δ0 trp1-Δ2 his3 lys2-128@ htb1-A84D(30, URA3) htb2-A84D(30, His3MX)	Tim Formosa
spt16-11; H2B A84D	MATa ura3 leu2 trp1 his3 lys2-128@ htb1-A84D(30, URA3) htb2-A84D(30, His3MX) spt16-11	Tim Formosa
WT-1 (*SPT16*) /p*STE5*-GFP		This study
spt16 E857K /p*STE5*-GFP		This study
WT-2(*SPT16*) /vector		This study

WT-2 (*SPT16*) /p*STE5*-GFP		This study
spt16-11/ p*STE5*-GFP		This study
spt16-11; A84D /p*STE5*-GFP		This study
WT-2 (*CLN2*-TAP)		This study
spt16-11 (*CLN2*-TAP)		This study
H2B WT (*SPT16*-GFP)		This study
H2B R95A (*SPT16*-GFP)		This study
H2B S93A		Ali Shilatifard
H2B R102A		Ali Shilatifard

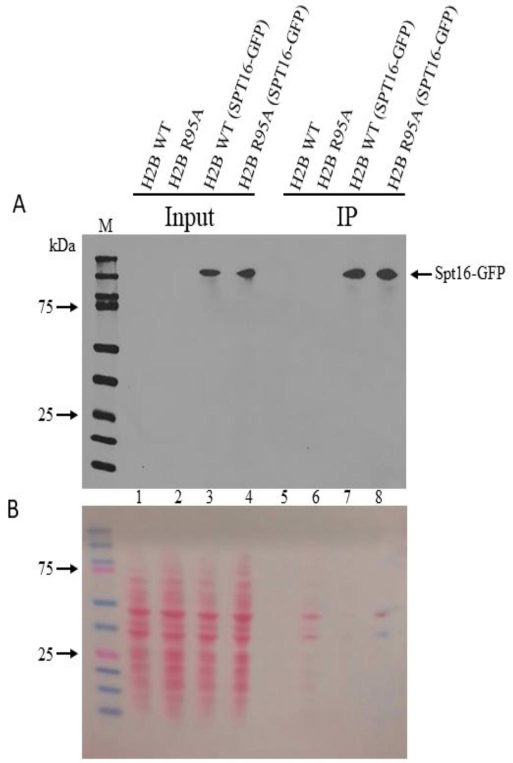

Figure S1. Independent experiment showing immunoblot detection of Spt16-GFP in the total extracts (input) and bound to the anti-GFP beads (IP). Spt16-GFP input, lanes 3 and 4, total extracts from H2B WT-FLAG and H2B R95A-FLAG cells expressing Spt16-GFP, respectively, and probed with anti-GFP. Spt16-GFP pull down by anti-GFP beads (IP), lanes 7 and 8.

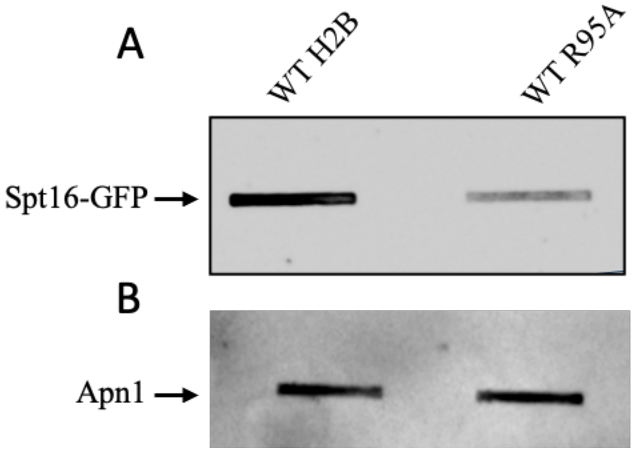

Figure S2. Spt16-GFP binds weakly to the chromosomal DNA of the H2B-R95A mutant as compared to WT. Briefly, cells were subjected to the RADAR assay (see Materials and Methods) and the DNA with bound proteins was quantified before processing on a slot-blot to detect Spt16-GFP and the control protein Apn1.

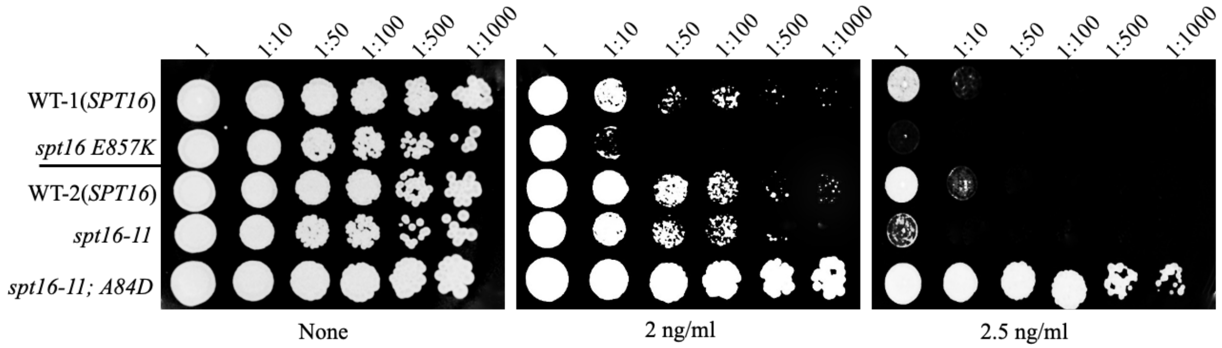

Figure S3. Spot test analysis showing the sensitivity and resistance of spt16 mutants towards rapamycin. The indicated WT and isogenic *spt16* mutants were grown overnight and the next day serially diluted and spotted onto plates without and with rapamycin. The plates were photographed 48 hrs following incubation at 30 ºC.

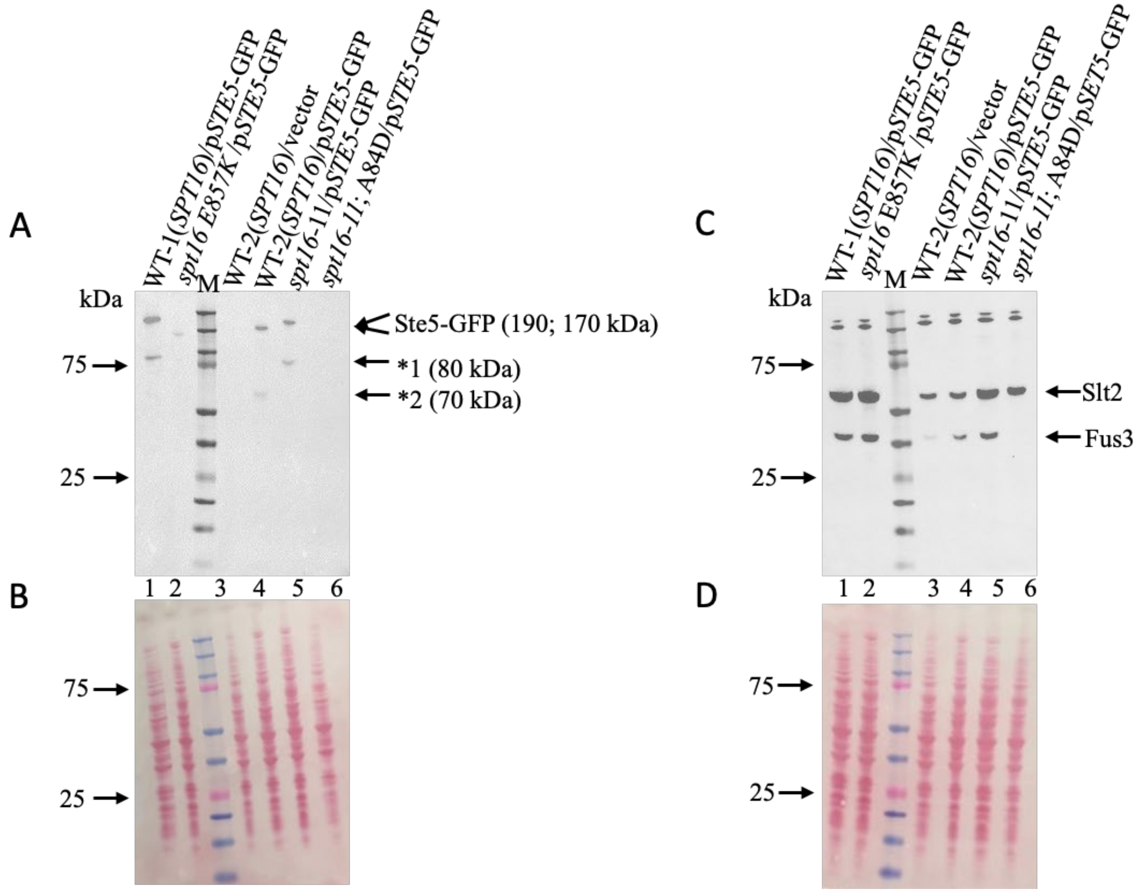

Figure S4. An independent experiment showing that *spt16* mutants expressed different forms and levels of Ste5-GFP. The experiment was carried out as indicated in the legend of Fig. 4, except the samples were analyzed using a gradient gel.

The histone H2B Arg95 residue efficiently recruits the transcription factor Spt16 to mediate Ste5 expression of the pheromone response pathway

Abdallah Alhaj Sulaiman[1], Reem Ali[1], and Dindial Ramotar[1, a]

Running title: Arginine 95 of H2B is essential for Spt16 recruitment

[1]Division of Biological and Biomedical Sciences, College of Health and Life Sciences, Hamad Bin Khalifa University, Education City, Qatar Foundation, Doha, Qatar, P.O.Box: 34110

[a] Corresponding author Email: dramotar@hbku.edu.qa

The following is the full raw data for the figures in the text.

Full raw data for Figure 1.

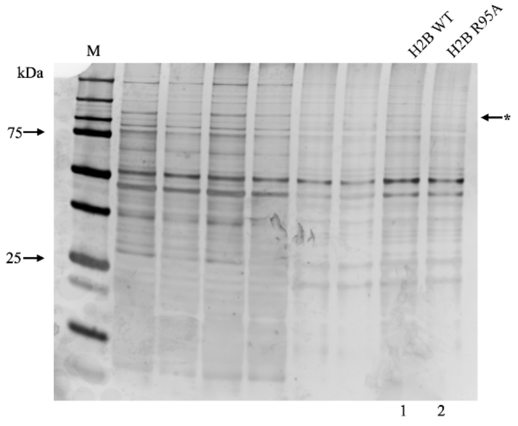

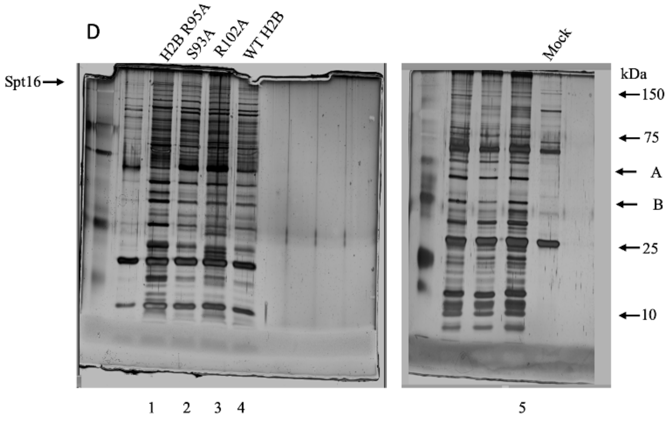

Full raw data for Figure 2.

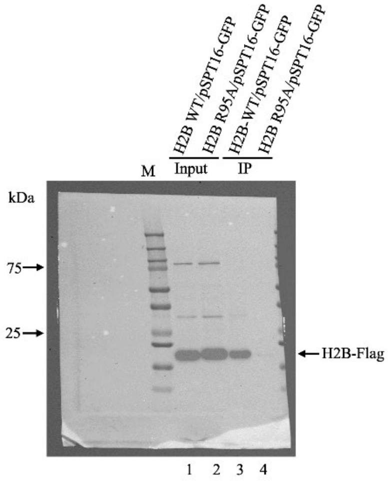

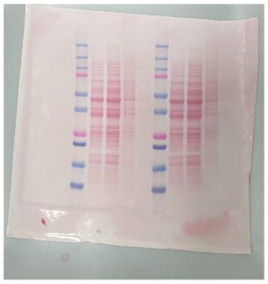

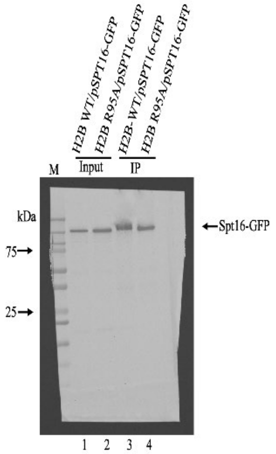

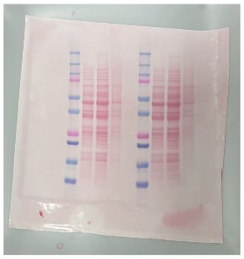

Full raw data for Figure 4.

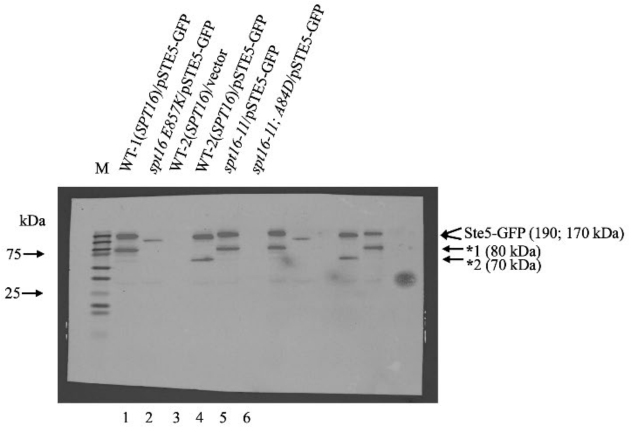

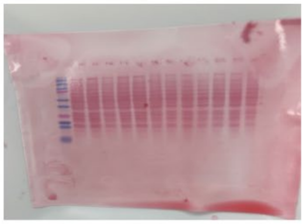

Full raw data for Figure 5.

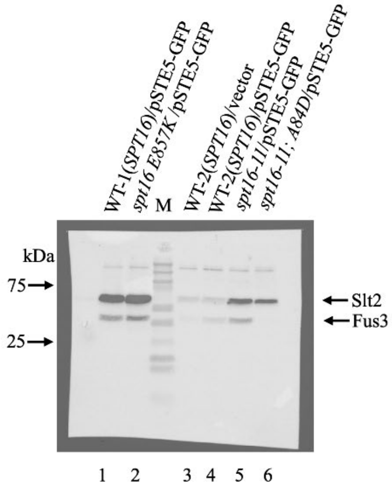

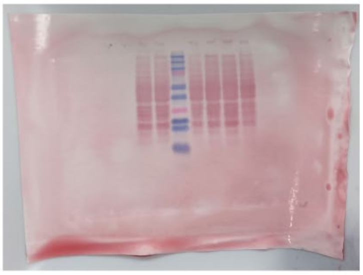

Full raw data for Figure 6.

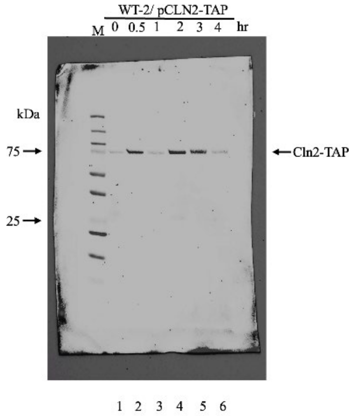

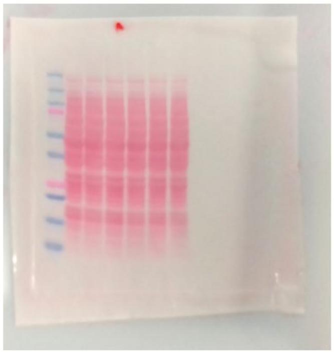

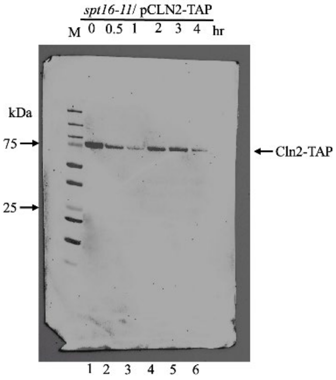

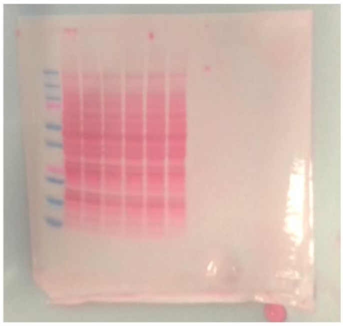

Full raw data for Figure S1.

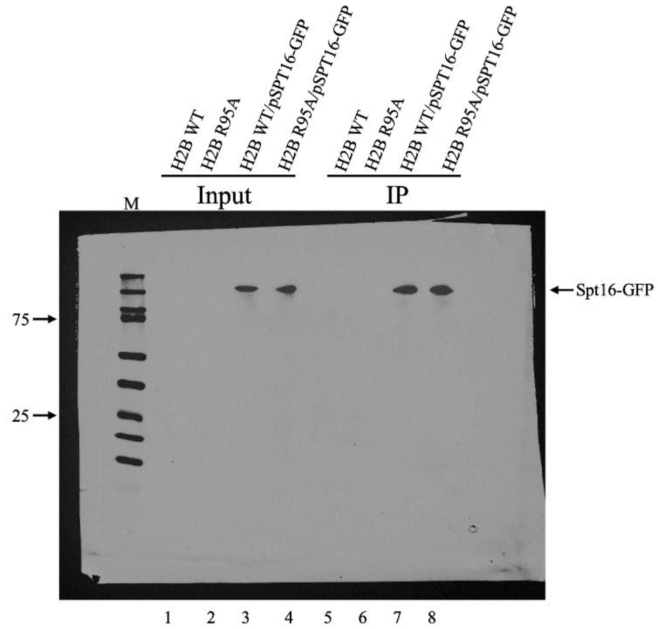

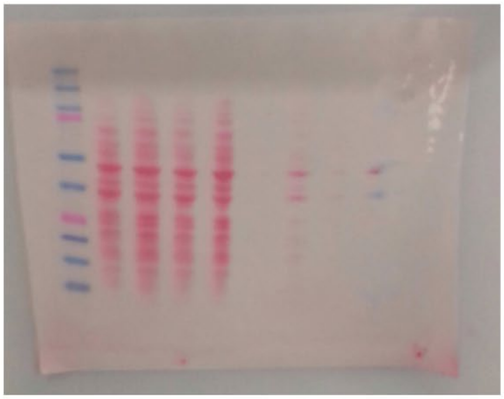

Full raw data for Figure S2.

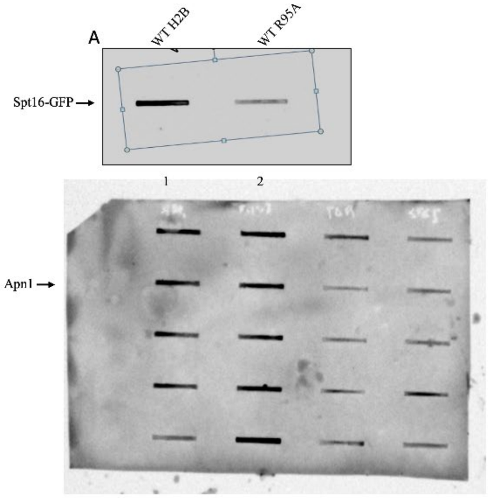

Full raw data for Figure S4.

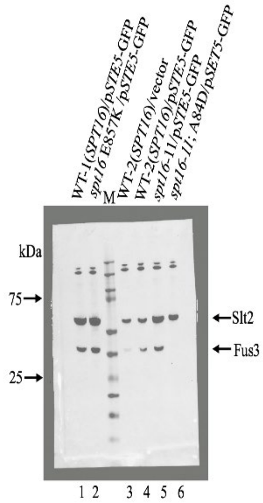

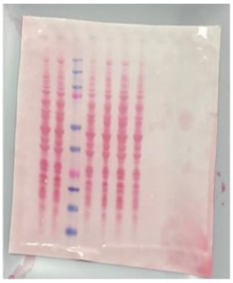

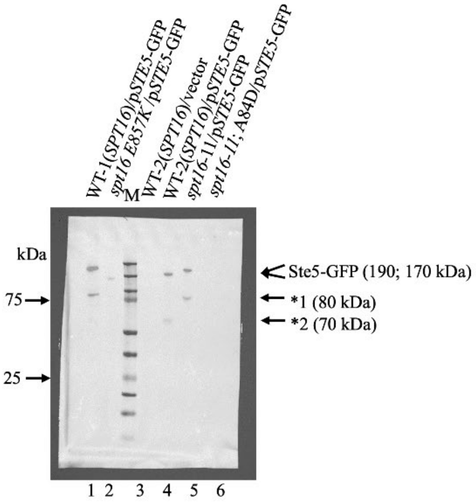

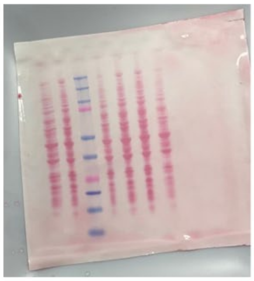

Supplement: Supplementary file 1 — Supplementary Information. [file 41598_2023_37339_MOESM1_ESM.pdf]
